# Difference in distant failure site between locally advanced squamous cell carcinoma and adenocarcinoma of the uterine cervix after C-ion RT

**DOI:** 10.1093/jrr/rru117

**Published:** 2015-01-14

**Authors:** Masaru Wakatsuki, Shingo Kato, Tatsuya Ohno, Hiroki Kiyohara, Kumiko Karasawa, Tomoaki Tamaki, Ken Ando, Daisuke Irie, Shintaro Shiba, Hirohiko Tsujii, Takashi Nakano, Tadashi Kamada, Makio Shozu

**Affiliations:** 1 Research Center for Charged Particle Therapy, National Institute of Radiological Sciences, 4-9-1 Anagawa, Inage-ku, Chiba 263-8555, Japan; 2 Department of Radiation Oncology, Saitama Medical University International Medical Center, Saitama, Japan; 3 Department of Radiation Oncology, Gunma University Graduate School of Medicine, Maebashi, Gunma, Japan; 4 Department of Radiation Oncology, Gunma Prefectural Cancer Center, Ota, Gunma, Japan; 5 Department of Reproductive Medicine, Graduate School of Medicine, Chiba University, Chiba, Japan

**Keywords:** carbon-ion radiotherapy, uterine cervical cancer, distant failure, adenocarcinoma of the uterine cervix, para-aortic lymph node metastasis

## Abstract

We investigated the first site of distant failure after carbon ion radiotherapy (C-ion RT) for locally advanced cervical cancer in three clinical trials. A total of 91 cases were enrolled in the three trials (Protocol 9702, 9704 and 9902). Histologically, 36 cases had squamous cell carcinoma (SqCC) and 55 cases had adenocarcinoma (AC), including 13 with adenosquamous cell carcinoma. The number of cases with Stage IIB, IIIB and IVA disease was 21, 59 and 11, respectively. Of the 91 cases, 42 had positive pelvic lymph nodes (PLNs). The median tumor size was 6.0 cm (range, 3.0–12.0 cm). The median follow-up duration for all cases was 40 months (range, 7–181 months). A total of 40 cases developed distant failure as the first site of failure: 13 of 36 (36.1%) SqCC cases had distant failure, with 9 of them with para-aortic lymph node (PALN) failure; 27 of 55 (44.0%) AC cases had distant failure, and 23 of them had distant failure excluding PALN metastasis. Distant failure rates of SqCC cases who had positive and negative PLNs before C-ion RT were 61.1% and 11.1%, respectively (*P* = 0.0045). Those of AC cases were 54.2% and 45.2%, respectively (*P* = 0.507). In conclusion, there were high rates of distant failure after C-ion RT in AC cases regardless of PLN status, and there were high rates of distant failure after C-ion RT, especially PALN failure, in SqCC cases with positive PLNs.

## INTRODUCTION

In 1994, carbon ion radiation therapy (C-ion RT) was initiated at the National Institute of Radiological Sciences (NIRS) [1]. Carbon ion beams have improved dose localization properties, and this potentiality can produce great effects on tumors while minimizing normal tissue damage. Moreover, they possess a biological advantage due to their high relative biological effectiveness in the Bragg Peak [1–4]. A number of reports have demonstrated the favorable results of C-ion RT in the treatment of malignant tumors [1, 5–9]. Our group has reported four clinical trials of C-ion RT for locally advanced cervical carcinoma in which C-ion RT achieved a relatively better local control rate than conventional treatment for bulky tumors [9–12].

Concurrent chemoradiation therapy (CCRT) is a standard therapy for locally advanced uterine cervical cancer. It is well known that a major failure site after CCRT is the pelvic area, such as local or pelvic lymph node (PLN) recurrence [13–17]. The distant metastasis rate is very high in cases with advanced disease, and the para-aortic lymph nodes (PALNs) are a major site of distant metastasis [18]. However, the pattern of distant metastases in cases with a bulky tumor or adenocarcinoma (AC) remains unclear, because such cases show higher rates of local failure and there will be included 2nd metastases when they have local failure.

In our clinical trials of C-ion RT, even though local control rates were relatively better than those of conventional studies for bulky tumor or AC, overall survival rates were still unsatisfactory because of frequent distant failure [10, 12, 19]. Thus, the current study analyzed the failure pattern of cervical cancer with bulky tumor or AC after C-ion RT, and it is presented as a supplementary analysis of three clinical trials (Protocol 9702, Protocol 9704 and Protocol 9902).

## MATERIALS AND METHODS

### Patient eligibility

Patients were enrolled into the studies if they had previously untreated squamous cell carcinoma (SqCC) (Protocol 9702 and Protocol 9902) or AC (Protocol 9704) of the uterine cervix with International Federation of Gynecology and Obstetrics (FIGO 1994) Stage IIB, IIIB or IVA disease, and without rectal invasion. The tumor had to be grossly measurable. Other eligibility criteria included World Health Organization performance status <3, age <80 years, and estimated life expectancy of >6 months. Patients with histories of prior chemotherapy or pelvic radiotherapy were excluded from the studies. Patients were also excluded if they had severe pelvic infection, severe psychological illness, or active synchronous cancer. Pretreatment evaluation consisted of an assessment of the patient's history, physical and pelvic examinations by gynecologists and radiation oncologists, a cervical biopsy, routine blood cell counts, a chemistry profile, a chest X-ray, cystoscopy and rectoscopy. Bladder or rectal involvement was assessed by the findings of endoscopy. CT scans of the abdomen and pelvis, magnetic resonance imaging (MRI) of the pelvis, and ^11^C methionine positron emission tomography (PET) scans were also performed for all patients. Patients were staged according to the FIGO staging system. Lymph node status was classified by the short axis on CT images as negative (<1 cm) or positive (≥1 cm), and patients with PALNs ≥ 1 cm in minimum diameter on CT images were excluded from the studies (although patients with enlarged PLNs only were included). The tumor size was assessed by both pelvic examination and MRI, and the dimensions of the cervical tumor were measured according to T2-weighted MRI images. ^11^C methionine PET scans were supplementally used for detecting distant metastases. Working group pathologists reviewed the tumor specimens.

### Carbon ion radiotherapy

The treatment by C-ion RT has previously been described [9–11]. Briefly, the treatment consisted of whole pelvic irradiation and local boost. Prophylactic whole pelvic irradiation included all areas of gross and potentially microscopic disease, consisting of the cervical tumor, uterus, parametrium, at least the upper half of the vagina, and PLNs (common iliac, internal iliac, external iliac, obturator and presacral lymph nodes). These three clinical trials were dose escalation studies for local boost, so total doses to the cervical tumor were 62.4–74.4 GyE in 20 or 24 fractions. Local failure in correlation with dose escalation has already been discussed in other reports [9–12]. Total dose for the whole pelvis was 44.8 GyE in 16 fractions (Protocol 9702), 39.0 GyE in 13 fractions (Protocol 9902), or 36.0 GyE in 12 fractions (Protocol 9704); none received concurrent chemotherapy and irradiation to the outside of the pelvis. The treatment protocol for the current study was reviewed and approved by the National Institute of Radiological Sciences Ethics Committee of Human Clinical Research, and all patients signed an informed consent form before the initiation of therapy.

### Assessment of failure patterns

After completion of C-ion RT, patients were followed up every 1–3 months for 2 years, and every 3 or 6 months thereafter. The first site of failure was evaluated in terms of locoregional (local and PLN) recurrence, PALN failure and distant failure. Distant failure included PALN failure. The cases of distant failure that occurred after locoregional recurrence were included in ‘locoregional failure’ and excluded from ‘distant failure’. They were defined according to the evidence of tumor regrowth or recurrence by physical examination, CT, MRI, PET and/or biopsy. The overall survival rate was calculated using the Kaplan–Meier method. The log-rank test and chi-square test were used for statistical analyses and performed with SPSS software, version 16.0.

## RESULTS

Treatment outcomes for each trial, such as local control rate, overall survival rate, acute toxicities and late toxicities, have already been reported [9–12]. The patient characteristics are summarized in Table 1. A total of 91 cases were enrolled in these clinical trials. Histologically, 36 cases had SqCC, and 55 cases had AC (including 13 cases with adenosquamous cell carcinoma). The number of cases with Stage IIB, IIIB and IVA disease was 21, 59 and 11, respectively. All cases with Stage IVA had bladder invasion but no rectal invasion. Of the 91 cases, 42 had enlarged PLNs. Most of the cases had a bulky tumor, and the median tumor size was 6.0 cm (range, 3.0–12.0 cm). Staging laparotomy was not performed, and no histologic confirmation of CT-positive PLNs or PALNs was obtained. No patient underwent lymph node resection. Overall treatment time (OTT) ranged from 31 to 47 days, with a median of 35 days. The median follow-up duration for all cases and surviving patients was 40 months (range, 7–181 months) and 126 months (range, 40–181 months), respectively. In the case of SqCC, the median follow-up duration for all patients and surviving patients was 37 months (range, 8–181 months) and 148.0 months (range, 105–181 months), respectively. In the case of AC, the median follow-up duration for all patients and surviving patients was 40 months (range, 7–159 months) and 72.0 months (range, 40–159 months), respectively.

**Table 1.** Patient characteristics

	SqCC cases	AC cases	Total
	(36 cases)	(55 cases)	(91 cases)
Stage			
IIB	1	20	21
IIIB	26	33	59
IVA	9	2	11
Pelvic LN			
Negative	18	31	49
Positive	18	24	42
Tumor size (median)	4.0–12.0 cm (6.4 cm)	3.0–11.8 cm (5.5 cm)	3.0–12.0 cm (6.0 cm)
Dose of C-ion RT (median)	64.0–72.8 GyE (68.8 GyE)	62.4–74.4 GyE (71.2 GyE)	62.4–74.4 GyE (71.2 GyE)
Overall treatment time (median)	31–47 days (35 days)	32–40 days (35 days)	31–47 days (35 days)

Table 2 shows the first site of failure after C-ion RT according to histology and PLN status or tumor size. Of the 91 cases, 27 developed locoregional recurrence and 40 had distant failure. In the case of SqCC, 9 of 36 (25.0%) cases had locoregional recurrence, 13 of 36 (36.1%) had distant failure, and 9 of 36 (25.0%) had PALN failure. In the case of AC, 18 of 55 (32.7%) developed locoregional recurrence, 27 of 55 (49.1%) had distant failure, and 10 of 55 (18.2%) had PALN failure.

**Table 2.** Sites of failure after C-ion RT (pelvic lymph node and tumor size)

		PLN		Tumor size		Total
	First site of failure	Negative	Positive	≤ Median	> Median	36
SqCC	Locoregional failure	5 (27.8%)	4 (22.2%)	4 (22.2%)	5 (27.8%)	9 (25.0%)
	Distant failure	2 (11.1%)	11 (61.1%)	6 (33.3%)	7 (38.9%)	13 (36.1%)
	PALN failure	1 (5.5%)	8 (44.4%)	4 (22.2%)	5 (27.8%)	9 (25.0%)
	Total	18	18	18	18	36
AC	Locoregional failure	7 (22.6%)	11 (45.8%)	7 (25.0%)	11 (37.9%)	18 (32.7%)
	Distant failure	14 (45.2%)	13 (54.2%)	11 (39.3%)	16 (59.3%)	27 (49.1%)
	PALN failure	2 (6.5%)	8 (33.3%)	4 (14.3%)	6 (22.2%)	10 (18.2%)
	Total	31	24	27	28	55

The distant failure rate of SqCC cases who were PLN-positive and -negative was 61.1% and 11.1%, respectively, demonstrating a significant difference (*P* = 0.0045). On the other hand, there was no significant difference between patients who were PLN-positive and -negative in the AC cases (54.2% and 45.2%, respectively; *P* = 0.508). There were no significant differences in the distant failure rate between relatively larger and smaller tumors in the SqCC cases (33.3% and 38.9%; *P* = 0.729) or in the AC cases (39.3% and 59.3%; *P* = 0.139) (Table 2). There were, however, significant differences in distant and PALN failure rates between 5 weeks and 6 weeks of treatment duration (*P* = 0.0014 and 0.0016), but there were no significant differences in distant and PALN failure rates between a total dose of less than 70 GyE and a total dose of more than 70 GyE (Table 3).

**Table 3.** Sites of failure after C-ion RT (treatment duration and total dose)

		Treatment duration		Total dose		Total
	First site of failure	5-week	6-week	≤70.0 GyE	>70.0 GyE	
SqCC	Locoregional failure	7 (36.4%)	1 (7.1%)	8 (34.8%)	0 (0%)	9 (25.0%)
	Distant failure	4 (18.2%)	9 (64.3%)	11 (47.8%)	3 (23.1%)	13 (36.1%)
	PALN failure	1 (4.5%)	8 (57.1%)	6 (26.9%)	3 (23.1%)	9 (25.0%)
	Total	22	14	23	13	36
AC	Locoregional failure	18 (32.7%)	0	6 (35.3%)	12 (31.6%)	18 (32.7%)
	Distant failure	27 (49.1%)	0	6 (35.3%)	21 (55.3%)	27 (49.1%)
	PALN failure	10 (18.2%)	0	4 (23.5%)	6 (15.8%)	10 (18.2%)
	Total	55	0	17	38	55

**Fig. 1.**
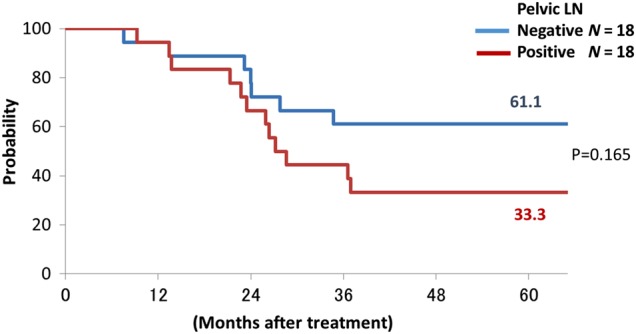
Overall survival curves in squamous cell carcinoma patients with and without pelvic lymph node metastases (pelvic lymph node negative: blue line; positive: red line).

**Fig. 2.**
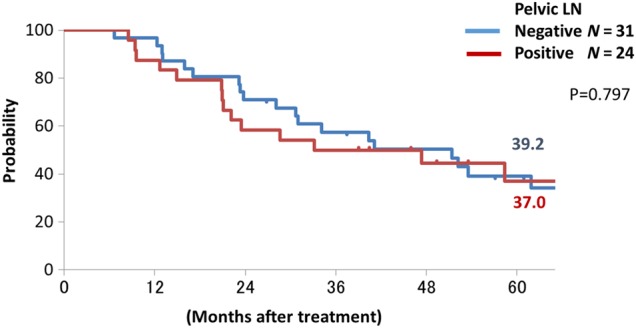
Overall survival curves in patients with adenocarcinoma with and without pelvic lymph node metastases (pelvic lymph node negative: blue line, positive: red line).

Figures 1 and 2 show the overall survival curves for SqCC and AC. The 5-year survival rates for SqCC cases with positive and negative PLNs were 33.3% and 61.1%, respectively (*P* = 0.165), and those of AC cases were 38.7% and 36.7%, respectively (*P* = 0.912).

Distant failure was the first site of failure for 13 of the SqCC cases and 27 of the AC cases, and the first sites of distant failure in SqCC cases and AC cases after C-ion RT are listed in Tables 4 and 5, respectively. Of the SqCC cases, nine cases (69.2%) showed PALN failure, two had other lymph node sites, two had lung, and three had other organs (bone, liver or kidney). The median duration until developing PALN relapse was 3 months (range 1–12 months). Of the AC cases, 10 (37.3%) had PALN failure, 12 (44.4%) lung, 6 (22.2%) peritoneal dissemination, 5 (18.5%) other lymph nodes (inguinal, axillary and supraclavicular), and 3 (11.1%) had other organs (bone or liver) (Table 5). 17 cases (63.0%) had distant failure excluding PALN.

**Table 4.** First sites of distant metastases in SqCC cases (9702, 9902)

	No.	Duration (median)	
PALN	9 (69.2%)	1–12 months (3 months)	
Other LN	2 (15.4%)	1.4, 72 months	axillary lymph node 1 supraclavicular node 1
Lung	2 (15.4%)	12, 33 months	
Other organs	3 (23.1%)	4–7 months (6 months)	bone 1, liver 1 kidney 1
Total cases	13

**Table 5.** First sites of distant metastases in AC cases (9704)

	No.	Duration (median)	
PALN	10 (37.3%)	1–43 months (6 months)	
Other LN	5 (18.5%)	1–15 months (7 months)	Inguinal lymph node 3 supraclavicular node 2
Lung	12 (44.4%)	3–52 months (8 months)	
Peritoneal dissemination	6 (22.2%)	5–37 months (15 months)	
Other organs	3 (11.1%)	6–43 months (19 months)	bone 2, liver 1
Total cases	27		

## DISCUSSION

The current study revealed that there were many distant failures excluding PALN failure after C-ion RT in AC cases, regardless of the PLN status, and there were high rates of PALN failure after C-ion RT in SqCC cases with bulky tumor and enlarged PLNs. In the current study, 27 of 55 (49.1%) AC cases had distant failure, and 17 of them (63.0%) had distant failure without PALN failure after C-ion RT. Eifel *et al*. reported the sites of distant failure in AC cases with Stage II, III and IV: 45.3% of the cases developed distant failure, but only 9.4% had PALN failure [20]. The current study showed similar results to their study, but they did not analyze the correlation between distant failure and PLN status. In the current study, even though 2 of 18 (11.1%) had distant failure in SqCC cases with negative PLNs, 14 of 31 (45.2%) had distant failure in AC cases with negative PLNs, and the distant failure rate of AC cases was significantly higher than that of SqCC cases in the cases with negative PLNs (*P* = 0.033). In addition, overall survival curves of AC cases with positive and negative PLNs showed similar curves (Fig. 2). These results suggested that locally advanced AC of the uterine cervix is a systemic disease (regardless of the PLN status) in comparison with SqCC. Recently we started another clinical trial of concurrent chemotherapy with C-ion RT for locally advanced AC of the uterine cervix.

In SqCC cases, the current study showed that there were high rates of PALN failure after C-ion RT in patients with bulky tumor and enlarged PLNs. Nelson *et al*. reported that PALN metastasis occurred in 14.9% of Stage IIB cases and 38.4% of Stage IIIB cases on the basis of PALN biopsies [18]. Eifel *et al*. reported a PALN failure rate at 5 years after CCRT of 7% in patients with locally advanced cervical cancer, with 47 of 195 patients with enlarged pelvic PLNs [21]. In the current study, the PALN failure rate was 25.0% of SqCC cases, relatively higher than that of Eifel's study, as the current study did not include surgical staging for PALN and had higher rates of patients with positive PLNs (50%). The distant failure rates of SqCC cases with positive and negative PLNs were 61.1% and 11.1%, respectively, an obviously significant difference (*P* = 0.0045). It is noteworthy that, in 9 of 13 cases with distant failure showing PALN failures, the median duration until developing PALN relapse was 3 months (range 1–12 months) (Table 4), and in fact several patients presented with PALN failure during or just after C-ion RT. These results suggested that prophylactic para-aortic irradiation for SqCC cases with positive PLNs might be beneficial. Thus, we are conducting a new clinical trial of prophylactic extended-field C-ion RT for locally advanced SqCC of the uterine cervix.

On the other hand, there were no correlation between distant failure and tumor size (Table 2). Morris *et al*. reported that the rates of distant failure after CCRT and RT alone were 14% and 33%, respectively [22]. Nakano *et al*. reported that the rate of distant failure after RT alone was 14% for Stage II disease and 25% for Stage III disease [23]. In cases with AC, Eifel *et al*. reported that 45% of cases with Stage IIB or III showed distant metastases after RT alone [20], and Huang *et al*. reported a 5-year distant failure rate of 46% for Stage III cases after RT alone or CCRT [24]. In the current study, the distant failure rates of SqCC cases and AC cases were 36.1% and 49.1%, respectively. These rates were similar to those of the other studies, despite the median tumor size being 6.0 cm (range, 3.0–12.0 cm) in the current study (larger than that of the other reports). Toita *et al*. reported a 2-year distant failure rate of 19% for tumors <50 mm, 20% for tumors 50–70 mm, and 47% for tumors >70 mm, also concluding that the incidence of distant failure increased with increased tumor size [15]. On the other hand, in our study there were no significant differences in distant failure rate between relatively larger and smaller tumors in the SqCC cases (33.3% and 38.9%, respectively; *P* = 0.729) and the AC cases (39.3% and 59.3%, respectively; *P* = 0.139) (Table 2). One of the possible reasons for this might be the reduction of spread to distant organs after the beginning of C-ion RT because of the shorter overall treatment time (OTT), because radiation may enhance the invasive and metastatic potential of cancer cells during overall treatment [25]. There were significant differences in distant failure rate between shorter and longer OTT groups (Table 3). OTT ranged from 45 to 60 days in most clinical trials for uterine cervical cancer by CCRT [26], but the median OTT for C-ion RT in these clinical trials was only 35 days, with C-ion RT achieving shorter OTT in a safe manner [12, 19].

The current study suffers from an important limitation, and that is the fact that there are as yet few C-ion RT facilities. However, higher local control rate and shorter OTT for bulky cervical cancer are attractive objectives, and they will be achieved by the use of high accuracy external radiation therapy and image-guided brachytherapy. Therefore, in the future, the results of this study may be expected to provide additional improvement.

In conclusion, there were high rates of PALN failure after C-ion RT in SqCC cases with enlarged PLNs, suggesting that prophylactic para-aortic irradiation for these cases might be beneficial. On the other hand, there were many distant failures after C-ion RT in AC cases, regardless of the PLN status; these cases would be classified as having systemic disease. Thus, therapeutic improvement for locally advanced AC might be expected from the use of concurrent chemotherapy, and we are now conducting new clinical trials for SqCC and AC.

## FUNDING

This work was supported by the Research Project with Heavy Ions at the National Institute of Radiological Sciences and Grant-in-Aid for Young Scientists B for Japan Society for the Promotion of Science Grant Number 26861026. Funding to pay the Open Access publication charges for this article was provided by Grant-in-Aid for Young Scientists B for Japan Society for the Promotion of Science Grant Number 26861026.
